# A novel *Bifidobacterium infantis*-mediated TK/GCV suicide gene therapy system exhibits antitumor activity in a rat model of bladder cancer

**DOI:** 10.1186/1756-9966-28-155

**Published:** 2009-12-16

**Authors:** Wei Tang, Yunfeng He, Shengcai Zhou, Yongping Ma, Geli Liu

**Affiliations:** 1 Department of Urology, the First Affiliated Hospital, Chongqing Medical University, Chongqing, China; 2 Department of Biochemistry and Molecular Biology, the School of Basic Medical Sciences, Chongqing Medical University, Chongqing, China

## Abstract

Bladder cancer is the ninth most common malignancy in the world. Successful clinical management remains a challenge. In order To search for novel targeted and efficacious treatment, we sought to investigate anti-tumor activity of BI-TK suicide gene therapy system in a rat model of bladder tumors. We first constructed and tested an anaerobic Bifidobacterium infantis-mediated thymidine kinase (BI-TK) suicide gene therapy system. To test the in vivo efficacy of this system, we established a rat model of bladder tumors, which was induced by N-methyl-nitrosourea perfusion. Bifidobacterium infantis containing the HSV-TK (i.e., BI-TK) were constructed by transformation of recombinant plasmid pGEX - TK. The engineered BI-TK was injected into tumor-bearing rats via tail vein, followed by intraperitoneal injection of ganciclovir (GCV). Using the rat model of bladder tumors, we found that bladder tumor burdens were significantly lower in the rats treated with BI-TK/GCV group than that treated with normal saline control group (*p *<*0.05*). While various degrees of apoptosis of the tumor cells were detected in all groups using in situ TUNEL assay, apoptosis was mostly notable in the BI-TK/GCV treatment group. Immunohistochemical staining further demonstrated that the BI-TK/GCV treatment group had the highest level of caspase3 protein expression than that of the empty plasmid group and normal saline group (p < 0.05). Thus, our results demonstrate that the Bifidobacterium infantis-mediated TK/GCV suicide gene therapy system can effectively inhibit rat bladder tumor growth, possibly through increasing caspase 3 expression and inducing apoptosis.

## Introduction

Bladder cancer is the ninth most common malignancy in the world. Current treatments for bladder cancer include surgery, immunotherapy, chemotherapy and radiotherapy. There is an increasing trend towards multimodal treatments. Although there have been substantial changes in the therapeutic options for the management of both superficial and muscle-invasive bladder cancer in the last 10 years, successful clinical management still posses a challenge for urologists and oncologists due to the high rate for recurrence and progression. It is conceivable that the efficacy of treatment may significantly be improved by targeted and/or advanced drug delivery strategies, which may result in increased treatment specificity together with lower toxic potential and higher therapeutic indices. Novel therapeutic modalities under investigation include DNA vaccines, magnetically targeted carriers, bio-adhesive microspheres and antisense oligodeoxynucleotides. For muscle-invasive bladder cancer, perioperative chemotherapy is used with increasing frequency. The latest preclinical research efforts are focused on the inhibition of angiogenesis and other processes predisposing to metastatic disease.

Cancer gene therapy is an important and promising area of cancer research. The development of a tumor-specific targeting tumor gene transfer system is the key to the success of gene therapy technique. It has been shown that Bifidobacterium infantis can specifically target the anaerobic tumor cells, and hence is a good tumor - targeting gene therapy vector system. Herpes Simplex Virus Thymidine kinase/ganciclovir (HSV-TK/GCV) system is currently one of the best studied tumor suicide gene therapy system. The thymidine kinase expressed specifically in tumor tissues can convert the non-toxic precursor ganciclovir into the ganciclovir-3-phosphate, a toxic substance that kills tumor cells. In this study, we developed and validated a novel suicide gene therapy system by exploring the hypoxic environment of solid tumors and the anaerobic metabolism features of Bifidobacterium infantis bacterial cells. Our results have demonstrated that the Bifidobacterium infantis/thymidine kinase suicide gene therapy system may be used as a targeted cancer therapy [1-5].

Currently animal models of bladder tumors are mostly limited to the use of xenograft tumor models with subcutaneous or planting bladder tumor cells. Subcutaneous xenograft tumor models are most commonly used because of many advantages, such as easy to establish and convenient to observe. However, these models neglect the anatomical and physiological characteristics of cancer-derived organs. In this study, we conducted MNU (methyl-nitroso-urea) reperfusion and induced rat bladder tumors with a high success rate. The morphological features and pathological features of the induced tumors are very similar to that of human bladder tumors, which come from the bladder epithelia. Histological examination confirmed that the induced tumors are transitional cell cancer in nature. MNU-induced bladder cancer seemingly has organ specificity. Thus, this method may represent an ideal approach to the development and treatment of bladder cancer [2,3]. Using this model, we investigated the *in vivo *efficacy of Bifidobacterium infantis-TK/GCV suicide gene therapy system in treating bladder tumors in rats. Our results have demonstrated that the Bifidobacterium infantis-mediated TK/GCV suicide gene therapy system can effectively inhibit rat bladder tumor growth via increasing caspase 3 expression and inducing apoptosis.

## Materials and methods

### Construction of the Bifidobacterium infantis -mediated TK/GCV suicide gene therapy system

Herpes simplex virus - thymidine kinase (HSV - TK) gene was PCR amplified and subcloned into pGEX-5X-1, at BamH I and Sal I sites (Takara, Tokyo, Japan), resulting in pGEX-TK. Potential recombinants were first screened by bacterial colony PCR, followed by DNA sequencing verification. After verification, pGEX-TK plasmid was used to transform electrocompetent Bifidobacterium infantis bacterial cells via electroporation, as previously reported [6-11].

### Experimental animals

Sprague-Dawley (SD) rats (6-8 weeks age, female, weighing 200-250 g) were housed at the Laboratory Animal Center of Chongqing Medical University, Chongqing, China. The animal experiments followed institutional guidelines for the use and care of animals. Animals were housed in microisolator cages under a specific pathogen-free (SPF) condition with 12-hour light-dark cycles.

### Bacterial strains and cultivation

*Bifidobacterium infantis *(Sangon, Shanghai, China) was provided by Molecular Biology Laboratory of Chongqing Medical University. Bifidobacterium infantis bacterial cells were inoculated in MRS (De Man, Rogosa and Sharpe medium) liquid medium, and grown in an anaerobic tank with a mixed-gas (80% N2, 10% CO2, 10% H2) at 37°C overnight.

### Establishment of a rat model of bladder cancer andexperimental groups

A rat model of bladder tumor was induced by using MNU (USA, Jersey, Sigma). Fifty four tumor-bearing SD rats were randomly divided into three groups: the normal saline group (n = 18), the Bifidobacterium infantis-pGEX-5X-1 (n = 18), and the Bifidobacterium infantis-pGEX-TK (i.e., BI-TK) group (n = 18). Each group was given tail vein injection of saline, Bifidobacterium infantis-pGEX-5X-1, or Bifidobacterium infantis-TK (containing 4.4 × 109 Bifidobacterium infantis), once every week for two weeks. Each group also received daily intraperitoneal injection of ganciclovir (GCV) (50 mg/kg, Merck, Darmstadt, Germany) for 14 days. On the 15th day after treatment, all rats were sacrificed by the overdose of ketamine (400 mg/kg) and xylazine (50 mg/kg) and necropsy was performed. Total weight of bladders was determined (see below). Tumor tissues were retrieved and embedded in 4% paraformaldehyde for hematoxylin-eosin (H & E) staining.

### Determination of bladder total weight

After the rats were sacrificed, the bladders were retrieved by severing the jugular, urethra near the bladder neck and double ureter close to bladder wall. The bladder anterior wall was opened for examining bladder tumor formation; and the liquid was dried with filter papers, The total weight of the bladder was then determined for all animals in the study.

### Apoptosis of bladder tumor cells determined by TUNEL assay

The TUNEL assay was carried out according to the manufacturer's instructions (TUNEL kit; Roche, Darmstadt, Germany). Apoptotic cells (approximately 100 cells/field for three non-overlapping fields) were counted. Apoptosis index was calculated as the percentage of apoptosis cells over total counted cells.

### Immunohistochemical staining of Caspase3 protein expression in bladder tumor cells

Immunohistochemical staining was conducted according to manufacturer's instructions (Zhongshan Golden Bridge Inc, Shanghai, China). The tumor sections were probed with a biotinylated anti-Caspase 3 antibody, followed by incubation with strapavidin-horseradish peroxidase. The presence of Caspase 3 protein was visualized by adding horseradish peroxidase substrate diaminobenzidine solution. The cells were counterstained with hematoxylin. Positively staining cells were documented under a light microscope and quantitatively analyzed by the Image-Pro Plus Analysis system (Olympus, Tokyo, Japan) from at least five high power fields. The average value of the intensity of positive staining was defined as positive reaction area/field area.

### Statistical analysis

All the experimental data were processed using the SPSS11.0 software. The number of samples of analysis of variance was determined by using SN-K method. α = 0.05.

## Results

### Construction of a novel Bifidobacterium infantis-mediated TK/GCV suicide gene therapy system

**Figure 1:**
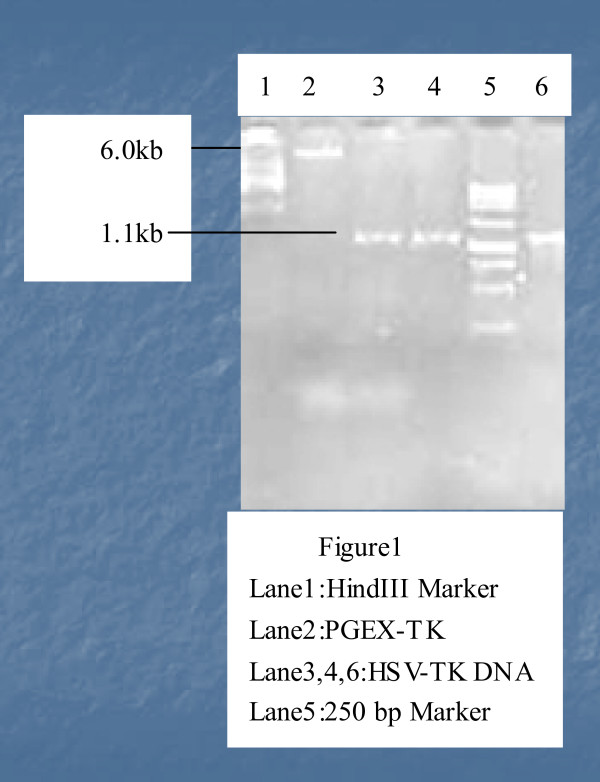
**Construction and verification of Bifidobacterium infantis-mediated TK tumor-targeting suicide gene therapy system**. Plasmid DNA was purified from anaerobic culture, digested with restriction enzymes, and resolved on 1% agarose gel. The expected 6.0 kb fragment of pGEX-TK is indicated by arrows.

The pGEX - TK recombinant vector was transformed into Bifidobacterium infantis by electroporation, After being cultured for 72 hours, Bifidobacterium infantis formed scattered colonies on the LB-plates containing MRS and ampicillin LB-plates. In contrast, transformatoion wild-type Bifidobacterium infantis only had no colonies on the MRS benzyl penicillin LB plates. Single colonies were picked up and grown under anaerobic condition. DNA was purified and verified by restriction enzymatic digestion and PCR amplification (Figure 1).

### Histologic analysis of rat bladder tumors

**Figure 2:**
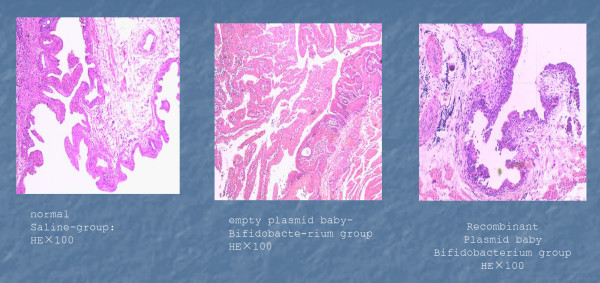
**Histologic evaluation of the MNU-induced rat bladder cancer**. MNU-induced bladder tumor samples were retrieved and subjected to paraffin-embedded sectioning and H & E staining. (A) Normal saline group, (B) Bifutobacterium infantis with empty plasmid group, and (C) Bifutobacterium infantis-PGEX-TK group. Representative samples are shown. Magnification, 100×.

As shown in Figure 2A, in the normal saline group tumors formed wide bases with short stalk structures. Tumor cells were highly heterogeneous, resembling the characteristics of human bladder cancers. Malignant cells were shown to infiltrate focal subtunica mucosa, muscular tunic. In both BI-pGEX-5X-1 and BI-pGEX-TK groups, the tumors grew much more slowly than that of the NS group; and tumor necrosis was more pronounced in these groups (Figures 2B and 2C).

### Significant reduction of the total weight of tumor-bearing bladders via BI-TK-mediated suicide gene therapy

As shown in Table 1, The bladder cancer occur in rat 9 weeks after MNU reperfusion, we used B-type ultrasonic inspection to measure the size of the tumor before treatment, the volume is no statistical significance. the total bladder weight of BI-TK group was significantly lower than that of the NS group (*p *<*0.01*). However, the weight difference between the NS group and the BI-pGEX-5X-1 group was not statistically different (*p *> *0.05*). These results suggest that the BI-TK/GCV tumor-targeting suicide gene therapy system may significantly inhibit bladder tumor growth.

**Table 1:** Bladder total weight of all tumor-bearing rat ( ± *s*, n = 18)

Groups	bladder total weight(mg)
NS group	302.33 ± 22.09
PGEX-5X-1- bifutobacterium infantis group	279.55 ± 21.17*
PGEX-TK- bifutobacterium infantis group	245.72 ± 13.34*

With regard to NS groups, *P < 0.05

Recombinant plasmid baby rat bladder bifidobacterium group was significantly lower than the total weight with the other groups, significant differences (p < 0.01). there was no significant difference between Saline group with empty plasmid baby Bifidobacterium group (p > 0.05); above results show that babies bifidobacterium - TK/GCV system gene targeting therapy can significantly inhibit bladder tumor growth.

### Detection of apoptosis in rat bladder tumors

**Figure 3:**
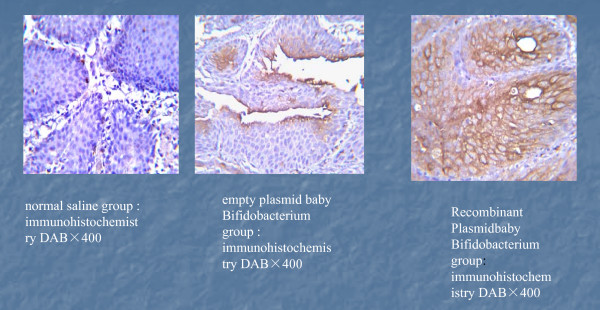
**Apoptosis analysis of BI-TK/GCV treated rat bladder cancer**. The TUNEL assay was carried out as described in Methods. Cells with positive staining were randomly counted in 10 high-power fields and the apoptosis index was calculated (mean SD). (A) Normal saline group (6.88 ± 1.40), (B) Bifutobacterium infantis with empty plasmid group (16.01 ± 3.48), and (C) Bifutobacterium infantis-PGEX-TK group (41.72 ± 4.27). There is statistically significant difference between each groups (p < 0.05). Representative samples are shown. Magnification, 100×.

Using the in situ TUNEL method, we found that each group exhibted varying degrees of apoptosis-staining positivity (Figure 3). The apoptotic indexes were 14.33 ± 5.29% for the NS group, 15.50 ± 4.34% for BI-pGEX-5X-1 group, and 29.44 ± 6.64% for BI-TK group, respectively. The apoptotic index for BI-TK group was significantly higher than that of BI-pGEX-5X-1 group or the NS group (*p *<*0.05*). These results indicate that BI-TK/GCV suicide gene therapy system can kill bladder cancer cells, possibly through inducing apoptosis.

### Caspase 3 protein expression in bladder tumor tissues

**Figure 4:**
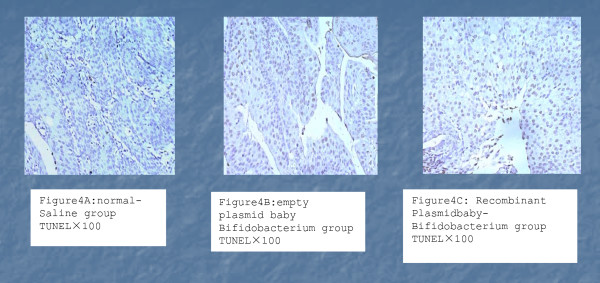
**Immunohistochemical staining of Caspase 3 expression in BI-TK/GCV treated rat bladder cancer**. The percentage of positive caspase 3 staining was 6.88 ± 1.40% for the normal saline group(A), 16.01 ± 3.48% for the BI-pGEX-5X-1 group(B), and 41.72 ± 4.27% for the BI-TK group(C), respectively. The differences between each group were statistically significant (*p *<*0.05*). ,100×.

We further analyzed the protein levels of caspase 3 in bladder tumor tissues by immunohistochemistry. Caspase 3 positive staining showed brownish yellow in the cytoplasm (in some cases, on cell membranes) (Figure 4). The percentage of positive caspase 3 staining was 41.72 ± 4.27% for the BI-TK group, 16.01 ± 3.48% for the BI-pGEX-5X-1 group, and 6.88 ± 1.40% for the normal saline group, respectively. The differences between each group were statistically significant (*p *<*0.05*). Nonetheless, these findings strongly suggest that BI-TK/GCV gene therapy system may upregulate Caspase 3 expression in bladder tumors and hence promote bladder tumor cell apoptosis (Figure 4).

## Discussion

Currently animal models of bladder tumors are mostly limited to the use of xenograft tumor models with subcutaneous or planting bladder tumor cells. Subcutaneous tumor model is most commonly used because of its easy manipulation, tumor growth consistency, and easy observation. However, the subcutaneous xenograft models ignore the anatomic and physiological characteristics of the organ. The method of MNU induce tumor have many good quality: easy, little used, induce way agility, it can be filling into bladder or injection by vein. Steinberg [12] evaluate the drug treatment therapeutic efficacy in MNU induced rat bladder tumor model, the result showed that the occurrence and biological behaviour is similar between MNU induced rat bladder tumor model and human TCCB, so MNU induced rat bladder tumor model can be used to research the treatment of bladder tumor.

In this study, we demonstrated that MNU reperfusion - induced rat bladder tumor have a high rate of success (nearly 100%) with morphological and pathological features similar to that of human bladder cancer. At the endpoint of this study, we also examined other organs, including liver, kidney, heart and lungs, and did not found any tumor formation, which is consistent with earlier reports [7,13-15]. Tumor-bearing rat was put to death l2 weeks after filling with MNU, cut open the bladder, we fond that the wall of bladder is thickening, pale and cauliflower like neoplasia, most of the bladder have many neoplasia, the serious one is full of neoplasia in the bladder. After 12 weeks, HE stain showed the typical TCCB (transitional cell carcinoma of the bladder) change appearance and focal under membrana mucosa, muscular layer infiltrate of tumor. It seems that the MNU bladder perfusion induced-cancer has organ specificity; and we did not find any adenocarcinoma or squamous cell carcinoma of the bladder histological changes. Therefore, MNU perfusion may represent an ideal approach for the establishment of animal models of bladder cancer for evaluating novel anti-cancer treatments.

Targeted cancer gene therapy is an ideal treatment for eradicating and/or limiting cancer growth and improving quality of life and survival rate of cancer patients. HSV-TK/GCV system is one of the most commonly used suicide gene therapy systems. However, most studies have used viral expression vectors, such as adenoviral or retroviral vectors to achieve the TK gene expression. Although efficient, these viral delivery systems have their own limitations, such as host immune response, low titer, the limited host range, serum complement inactivation, and detrimental mutations caused by random integrations into the host genome [3,16-19].

In this study, we explored the possible use of Bifidobacterium infantis as a tumor-targeting gene delivery vehicle in bladd cancer gene therapy. Bifidobacterium infantis are gram-positive bacteria which are non-pathogenic and strictly anaerobic without internal and external toxin production. It has been reported that Bifidobacterium can inhibit tumor growth [9,15,20]. Yazawa et al confirmed that when mammary tumors induced in rats were injected with Bifidobacterium via the tail vein, Bifidobacterium could propagate specifically in tumor tissuesproliferation, resulting in tumor tissue atrophy and extending the survival of tumor-bearing rats [9,15,20]. It has also been reported that when Bifidobacterium expressing human endostatin were injected to tumor-bearing mice via the tail vein, the antitumor effect was improved than the prototype Bifidobacterium [5,17,19]. These reports indicate that *Bifidobacterium *can be used as a tumor-targeting vector for cancer gene therapy [2-5,21]).

We have demonstrated the successful use of a novel Bifidobacterium infantis-mediated tumor-targeting suicide gene therapy system in inhibiting bladder tumor growth. Our results also indicate that induced apoptosis may at least in part account for the anticancer activity of the BI-TK system. Apoptosis, also known as programmed cell death, refers to certain physiological or pathological conditions in which the end of active life is regulated by the activation of a set of apoptotic factors. In normal cells, apoptosis and proliferation coexist and maintain a dynamic equilibrium. When the BI-TK/GCV suicide gene targeting system was delivered into tumor-bearing rats, we found that the system can significantly inhibit rat bladder tumor growth, induce apoptosis in tumor cells, and increase Caspase 3 protein. Therefore, our findings strongly suggest that Bifidobacterium infantis-mediated tumor-targeting suicide gene therapy system may represent a novel therapy for bladder cancer.

## Competing interests

The authors declare that they have no competing interests.

## Authors' contributions

WT, YH, SZ, YM, GL carried out the experiments described in the study. The Bifidobacterium infantis -mediated TK/GCV suicide gene therapy system is constructed by WT and YH. Bacterial strains and cultivation is finished by SZ and GL. Experimental of rat model finished by YM and WT. Apoptosis and Immunohistochemical is finished by WT and YH. Statistical analysis is finished by WT and YH. All authors read and approved the final manuscript.
